# Candidate Biopolymer Composite Membranes for Carbonic Anhydrase Immobilization in Enzymatic Direct Air Capture

**DOI:** 10.3390/ma19132869

**Published:** 2026-07-05

**Authors:** Spas Kerimov, Victoria Atanassova, Georgi Yankov, Radostin Stefanov, Ekaterina Iordanova, Georgi Marinov, Hristo Kalaydzhiev, Albert Krastanov

**Affiliations:** 1 Georgi Nadjakov Institute of Solid State Physics, Bulgarian Academy of Sciences, 72 Tzarigradsko Chaussee Blvd., 1784 Sofia, Bulgaria; vatanassova@issp.bas.bg (V.A.); eiordanova@issp.bas.bg (E.I.); 2 Institute of Optical Materials and Technologies “Acad. J. Malinowski”, Bulgarian Academy of Sciences, Akad. G. Bonchev Str., bl. 109, 1113 Sofia, Bulgaria; gmarinov@iomt.bas.bg; 3 Department of Analytical Chemistry and Physical Chemistry, Technological Faculty, University of Food Technologies, 26 Maritsa Blvd, 4002 Plovdiv, Bulgaria; 4 Department of Microbiology and Biotechnology, Technological Faculty, University of Food Technologies, 26 Maritsa Blvd., Marasha, 4002 Plovdiv, Bulgaria; a_krastanov@uft-plovdiv.bg

**Keywords:** direct air capture, carbonic anhydrase, EDC/NHS, chitosan, shellac, agarose, cellulose acetate, enzymatic membrane, biopolymer composite, CO_2_ hydration

## Abstract

**Highlights:**

- Chitosan/shellac membranes showed the strongest chemical–mechanical balance among screened candidate supports.
- Lyophilization preserved porous chitosan particle morphologies.
- FTIR spectra supported EDC/NHS-associated functional-group changes in reactive chitosan- and shellac-containing membranes.
- Reactive biopolymer membranes provide candidate supports for enzymatic DAC interfaces.
- Membrane selection requires balancing dry-state mechanics, hydration potential and reactive functionality.
- Chitosan/shellac is identified as the lead candidate for subsequent CA loading as an enzymatic carrier.

**Abstract:**

Direct air capture (DAC) requires carbon capture interfaces that operate under highly dilute CO_2_ conditions while minimizing thermal and chemical regeneration penalties. Carbonic anhydrase (CA) accelerates the reversible hydration of CO_2_ to bicarbonate and is therefore a strong biocatalytic candidate for low-temperature CO_2_ capture, but its implementation depends on candidate support materials that combine wet-state accessibility, chemical reactivity, mechanical processability and compatibility with membrane architectures. This study reports the preparation and screening of N-(3-dimethylaminopropyl)-N′-ethylcarbodiimide hydrochloride/N-hydroxysuccinimide (EDC/NHS)-reactive biopolymer composite membranes for future carbonic anhydrase (CA) immobilization. Chitosan particles were precipitated with citrate or tripolyphosphate under high-shear homogenization and compared after lyophilization or convective drying. Chitosan-, shellac-, agarose- and cellulose-acetate-based films plasticized with glycerol and/or polyethylene glycol 400 (PEG-400) were then evaluated by optical microscopy, dry-state penetrometric puncture testing, qualitative EDC/NHS-reactivity mapping and Fourier-transform infrared spectroscopy (FTIR). Freshly precipitated chitosan particles showed dendrite-like high-surface morphologies, while lyophilization preserved porous flocculated aggregates and convective drying produced denser collapsed structures. Neat chitosan showed the highest dry-state puncture force (2.230 ± 0.173 N), whereas chitosan/shellac (0.377 ± 0.044 N) and agarose/chitosan/PEG-400 (0.386 ± 0.038 N) provided the strongest reactive-composite compromise between dry-state puncture resistance and EDC/NHS compatibility. The EDC/NHS reactivity map identified chitosan- and shellac-containing films as the chemically most relevant supports because they provide amine and/or carboxyl functionality, whereas agarose and cellulose acetate alone were not directly suitable for zero-length amidation. FTIR spectra confirmed polymer-specific functional signatures and EDC/NHS-associated changes in carbonyl, amide and C-O/C-O-C regions, especially in shellac- and chitosan-containing composites. The results identify chitosan/shellac as the lead candidate membrane and agarose/chitosan/PEG-400 as a hydration-rich comparator for subsequent carbonic anhydrase immobilization studies. This work should be interpreted as a first-stage materials-screening study of candidate membranes for enzyme immobilization.

## 1. Introduction

Atmospheric CO_2_ accumulation and the continuing rise in greenhouse-gas concentrations require mitigation strategies that combine rapid emission reduction with carbon-dioxide-removal routes able to address dispersed and legacy emissions [1,2]. Direct air capture (DAC) is particularly relevant because it can remove CO_2_ independently of point-source location, but the low partial pressure of atmospheric CO_2_ imposes demanding mass-transfer and regeneration constraints on both liquid and solid sorbent systems [3,4]. Process analyses further show that energy demand and cost remain central barriers for DAC deployment, especially when dilute atmospheric CO_2_ must be separated into a concentrated product stream [5,6].

Membrane-based DAC has therefore attracted attention as a route toward continuous, modular capture architectures in which gas separation, liquid contact, sorbent regeneration and process monitoring may be distributed across different functional layers. Fujikawa et al. showed that very high membrane permeance could make multistage membrane-based DAC conceptually feasible under appropriate pressure-ratio and selectivity conditions [7]. Subsequent studies have framed DAC as a distinct and demanding membrane application, rather than a simple extension of flue-gas separation [8,9].

The design of candidate enzymatic DAC membranes can also be informed by mixed-matrix and hybrid membranes, where transport behavior is controlled by interfacial compatibility, dispersed-phase morphology, hydration domains, tortuosity and preferential transport channels. Recent work on MXene-polymer hybrid membranes for water purification emphasizes that interface design, functional regulation and channel architecture determine whether composite membranes improve transport or introduce defects and nonselective pathways [10]. This framework is relevant to chitosan/shellac and chitosan/agarose systems because future enzymatic DAC supports must combine reactive coupling sites, hydrated accessibility and mechanically continuous membrane morphology.

A subset category of membrane-based DAC is the enzymatic DAC approach, where a biological catalyst provides the direct-conversion site within a material or hydrated microenvironment and accelerates atmospheric CO_2_ uptake. Carbonic anhydrases (CAs) are particularly relevant because they are zinc metalloenzymes that catalyze the reversible hydration of CO_2_ to bicarbonate and a proton [11]. The catalytic mechanism depends on metal-bound hydroxide formation and efficient proton transfer, which explain the exceptionally high turnover of many alpha-class CAs [12,13,14]. Although carbonic anhydrase is widely studied in physiology and pharmacology, its catalytic chemistry is directly relevant to carbon capture because aqueous carbonate formation is often kinetically limited under mild conditions [15]. Several studies demonstrate that CA can accelerate CO_2_ absorption or support CO_2_ capture in liquid and membrane-contacting systems. Directed evolution has produced ultrastable CA variants for carbon-capture environments [16]. Earlier sequestration and absorption studies showed that free or immobilized CA can promote CO_2_ hydration in carbonate-containing solutions [17,18]. Hollow-fiber membrane bioreactors and enzymatic liquid membranes further demonstrate how CA can be positioned at gas-liquid or membrane-liquid interfaces for CO_2_ removal [19,20].

A decisive materials challenge is enzyme immobilization rather than maintaining high catalytic activity. Free CA can show high catalytic performance, but process implementation requires retention on a support carrier, suppression of leaching, preservation of water-rich active-site access, mechanical stability during operation and sufficient transport access to CO_2_ and bicarbonate transport pathways. These requirements make the support carriers as important as the enzyme itself. EDC/NHS chemistry is a common direct coupling route: EDC activates carboxyl groups through O-acylisourea intermediates, while NHS or sulfo-NHS stabilizes the activated intermediate as an amine-reactive ester, improving coupling efficiency and reducing uncontrolled hydrolysis [21,22,23]. Because hydrolysis competes with coupling, the availability and local accessibility of carboxyl and amine groups are critical [24]. For protein immobilization, polymer selection must be guided by functional-group availability rather than by film formation alone.

Chitosan is a logical component for enzymatic membrane supports because it is film-forming, hydrophilic, rich in primary amines and capable of ionic gelation with multivalent counterions [25]. The morphology of chitosan particles can be controlled by ionic-gelation chemistry; TPP-crosslinked systems are especially sensitive to pH, mixing and crosslinker concentration [26,27]. Preparation protocols also influence particle size and dispersity, which are relevant when particles are incorporated into films or used to increase surface accessibility [28].

Agarose, cellulose acetate and shellac provide complementary functions. Agarose contributes a hydrated polysaccharide network and can improve the water-compatible microenvironment of composite films, although unmodified agarose is not a strong EDC/NHS-reactive component [29,30]. Cellulose acetate is a processable membrane-forming polymer and can be combined with chitosan to tune film structure, but its ester-rich backbone is not a primary zero-length amidation partner [31,32]. Shellac is a natural resin containing carboxyl-bearing components, making it useful as a reactive co-component where EDC/NHS activation of carboxyl groups is desired [33,34]. Cellulose acetate can be used as a structural membrane phase, while chitosan and shellac supply the principal amine- and carboxyl-bearing domains relevant to EDC/NHS chemistry.

Polymers, such as poly(acrylic acid), poly(methacrylic acid) and polyamide-based materials, can provide higher densities of carboxyl or amine groups and may therefore support higher covalent enzyme-loading capacity. However, if the desired membrane systems are designed around bio-based or biopolymer-compatible components that could combine mild processing, hydrated enzyme compatibility, film formation and reactive functionality, these materials are not primary candidates. Future work should compare these bio-based candidate membranes against PAA-, PMAA- and polyamide-containing benchmark supports once carbonic anhydrase loading, retained activity and immobilization efficiency are directly evaluated.

Plasticizers are necessary because dry polysaccharide and cellulose-derivative films are often brittle. Glycerol and PEG-type plasticizers can increase flexibility by altering hydrogen bonding and chain mobility, but excessive plasticization can lower puncture resistance and cohesive strength [35,36,37]. Drying route is another central variable: freeze-drying can preserve porous ice-templated structures, whereas convective drying often produces capillary shrinkage, collapse and densification [38,39].

**Figure 1:**
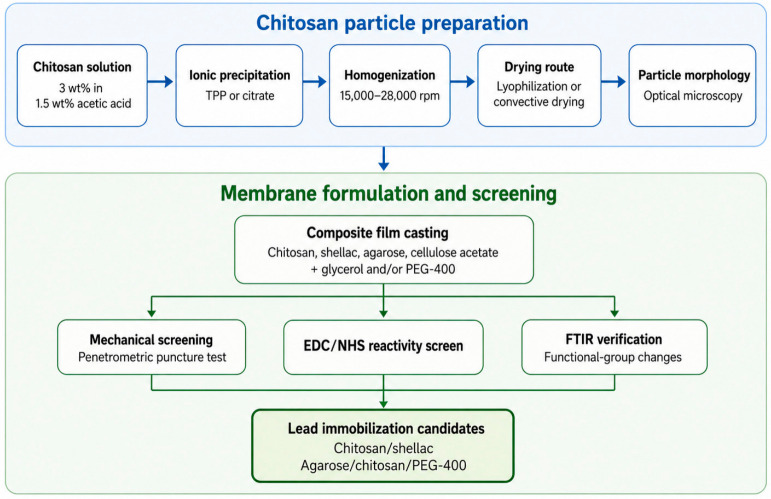
Experimental and conceptual workflow for screening biopolymer membranes as candidate CA immobilization supports.

This study reports the preparation, optical-morphological assessment, dry-state mechanical screening and chemical characterization of biopolymer composite membranes as candidate supports for future carbonic anhydrase immobilization in enzymatic DAC (Figure 1). The hypothesis is that chitosan- and shellac-containing composites will provide the most defensible EDC/NHS-reactive interfaces, while agarose and cellulose acetate will act primarily as structural or hydration-modifying components unless combined with a reactive partner.

## 2. Materials and Methods

### 2.1. Materials

For membrane preparation and chemical modification, the following materials were used: cellulose acetate (Mw ≈ 50,000 g mol^−1^ by GPC), low-molecular-weight chitosan, sodium tripolyphosphate (TPP), sodium citrate dihydrate, N-hydroxysuccinimide (NHS), N-(3-dimethylaminopropyl)-N′-ethylcarbodiimide hydrochloride (EDC), glycerol (molecular biology grade, ≥99.0%), PEG-400, low-EEO agarose, bleached and dewaxed shellac, glacial acetic acid, glutaraldehyde solution, sodium hydrogen phosphate and disodium hydrogen phosphate. All chemicals were purchased from Sigma-Aldrich (Darmstadt, Germany) and used without further purification. Silicone molds with a diameter of 4 cm were used for solvent casting of the films. Distilled water was used for the preparation of all aqueous solutions. The investigated polymer systems were selected to combine amine-containing polysaccharides (chitosan), carboxyl-containing resin components (shellac), hydrated polysaccharide matrices (agarose) and membrane-forming structural polymers (cellulose acetate) within composite architectures screened as candidate supports for future EDC/NHS-mediated immobilization of carbonic anhydrase. The functional role of the individual membrane components used in the present study is summarized in Table 1.

### 2.2. Preparation of Chitosan Particles

A 3 wt% chitosan solution was prepared in 1.5 wt% acetic acid using distilled water. Chitosan particles were precipitated in 25 mM sodium citrate, 2 wt% sodium tripolyphosphate (TPP) or 2 wt% glutaraldehyde prepared in phosphate buffer (pH 7.0–7.4). Homogenization was performed using a Fisherbrand™ 850 homogenizer (Fisher Scientific, Pittsburgh, PA, USA) equipped with a stainless-steel probe (10 mm diameter, 195 mm length, working volume 1.5–100 mL). Particle suspensions were processed at homogenization speeds between 15,000 and 28,000 rpm, depending on the formulation. Freshly prepared particles were characterized by optical microscopy prior to drying. Selected samples were subsequently dried either by convective drying or lyophilization for comparative morphological analysis. The precipitation media and homogenization parameters were selected based on the known sensitivity of chitosan particle morphology, aggregation state and surface structure to ionic-gelation chemistry and shear conditions [26,27,28].

### 2.3. Preparation of Polymer Films

**Figure 2:**
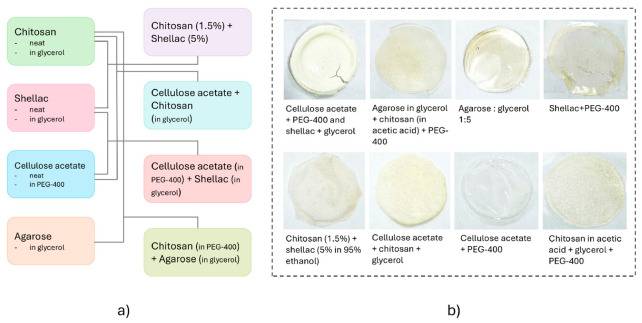
(**a**) Formulation map of the investigated biopolymer membrane systems, including neat, plasticized and blended polymer formulations. (**b**) Representative photographs of selected dried polymer membranes obtained from different composite formulations and further used for mechanical and FTIR screening.

Solvent-cast polymer films were prepared using chitosan, agarose, cellulose acetate, and shellac as single-component or composite systems. Glycerol and/or PEG-400 were used as plasticizers depending on the formulation. Chitosan films were prepared from chitosan dissolved in aqueous acetic acid, either neat or with glycerol and/or PEG-400. Agarose films were prepared with glycerol and, for composite systems, combined with chitosan and PEG-400. Cellulose acetate was dissolved in acetone and plasticized with PEG-400, while shellac was dissolved in 95% ethanol and combined with glycerol or PEG-400 where appropriate. Composite systems were prepared by mixing the individual polymer solutions under continuous stirring before casting into silicone molds (4 cm diameter). Selected formulations were additionally homogenized by short ultrasonic treatment (1–2 min) to improve particle dispersion within the polymer matrix. The investigated formulations included neat chitosan, plasticized chitosan, cellulose acetate/PEG-400, agarose/glycerol, chitosan/shellac, cellulose acetate/chitosan/glycerol, agarose/chitosan/PEG-400 and cellulose acetate/PEG-400/shellac/glycerol systems. Films were dried at 40 °C until complete solvent evaporation. A schematic overview of the prepared formulations and representative examples of the obtained membranes is presented in Figure 2, while the detailed compositions of the investigated systems are summarized in Supplementary Table S1. Preliminary experiments involving alginate- and shellac-rich systems were not included in subsequent analyses because the obtained films showed insufficient mechanical stability, excessive brittleness or strong adhesion to the casting molds.

### 2.4. Drying Procedures

Prepared chitosan particles and polymer films were dried either by convective drying or by lyophilization. Convective drying was performed using a SLW 115 SMART laboratory oven (POL-EKO-APARATURA, Wodzisław Śląski, Poland), while freeze-drying was carried out using a Modulyo lyophilizer (Edwards, West Sussex, UK). For film preparation, drying was typically performed at 40 °C under forced-convection conditions until complete solvent evaporation. Lyophilization and convective drying were compared because water removal by sublimation can preserve ice-templated pores, whereas evaporative drying can generate capillary stress, shrinkage and densification in hydrated polymer networks.

### 2.5. Optical Microscopy

Morphological observations of freshly prepared and dried chitosan particles were performed using an Olympus CX43 optical microscope (Evident Corporation, Tokyo, Japan). Micrographs were acquired through the eyepiece at magnifications of 10× and 40× depending on the sample morphology and aggregation state. The microscopy data were used for qualitative comparison of particle and aggregate morphology after different precipitation, homogenization and drying conditions.

### 2.6. Mechanical Characterization

Mechanical screening of the prepared films was performed in the dry state using a TX-700 texture analyzer (Lamy Rheology, Champagne au Mont d’Or, France) equipped with a 25 kg load cell. Penetrometric measurements were carried out using a blunt stainless-steel cylindrical probe with a diameter of 2 mm. The penetration speed was set to 0.5 mm s^−1^ and the penetration depth to 5 mm. The measured response parameter was puncture force. Neat chitosan films were tested using an increased maximum instrument force of 5 N because the standard 1 N setting was insufficient to penetrate the material. All other film formulations were tested under the standard 1 N force limit. The average film thickness used for mechanical testing was 1.5 ± 0.031 mm. These dry-state measurements were intended as a conservative first-stage stress screen of film integrity, brittleness and handling resistance after drying and processing.

### 2.7. EDC/NHS Activation

**Figure 3:**
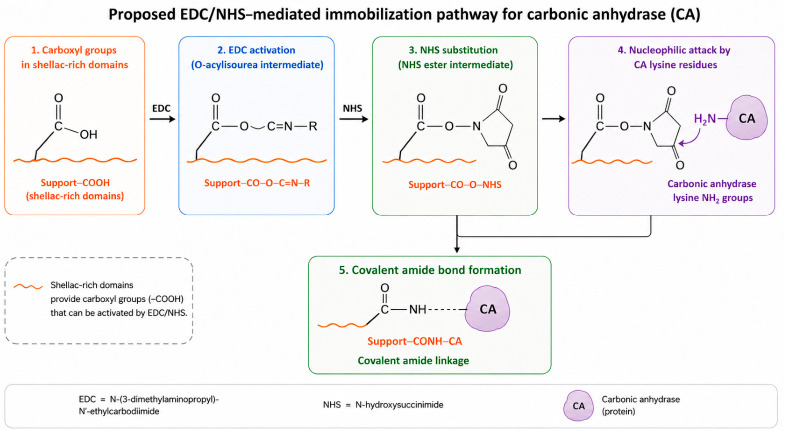
Proposed EDC/NHS-mediated coupling route for carbonic anhydrase immobilization on reactive biopolymer membrane supports.

Selected polymer films were chemically modified using EDC/NHS activation. The modification was performed to evaluate the suitability of the prepared polymer systems as candidate supports for subsequent carbonic anhydrase immobilization. Shellac-rich domains were considered particularly suitable for EDC/NHS-mediated activation because of the presence of carboxyl-containing functionalities, while chitosan-rich matrices provide accessible amine groups and hydrated polysaccharide regions potentially favorable for immobilization chemistry. The proposed coupling route within the investigated membrane systems is schematically illustrated in Figure 3.

### 2.8. FTIR Spectroscopy

Fourier-transform infrared spectroscopy (FTIR) was performed on both modified and non-modified polymer films to evaluate changes in the characteristic functional groups after EDC/NHS treatment. Measurements were carried out in attenuated total reflection (ATR) mode using a VERTEX 70 FTIR spectrometer (Bruker Optics, Ettlingen, Germany). Spectra were recorded in the 4000–600 cm^−1^ spectral range with a resolution of 2 cm^−1^ using 32 scans per sample. The obtained spectra were analyzed in the regions corresponding to O–H/N–H stretching (3600–3000 cm^−1^), aliphatic C–H stretching (3000–2850 cm^−1^), carbonyl vibrations (1750–1700 cm^−1^), amide-associated bands (1650–1550 cm^−1^) and C–O/C–O–C fingerprint vibrations (1300–900 cm^−1^). Band assignments followed established FTIR interpretations for chitosan, agarose, cellulose acetate, shellac and polysaccharide blends [40,41,42,43].

## 3. Results

### 3.1. Chitosan Particle Morphology Is Controlled by Counterion Chemistry, Homogenization and Drying Route

**Figure 4:**
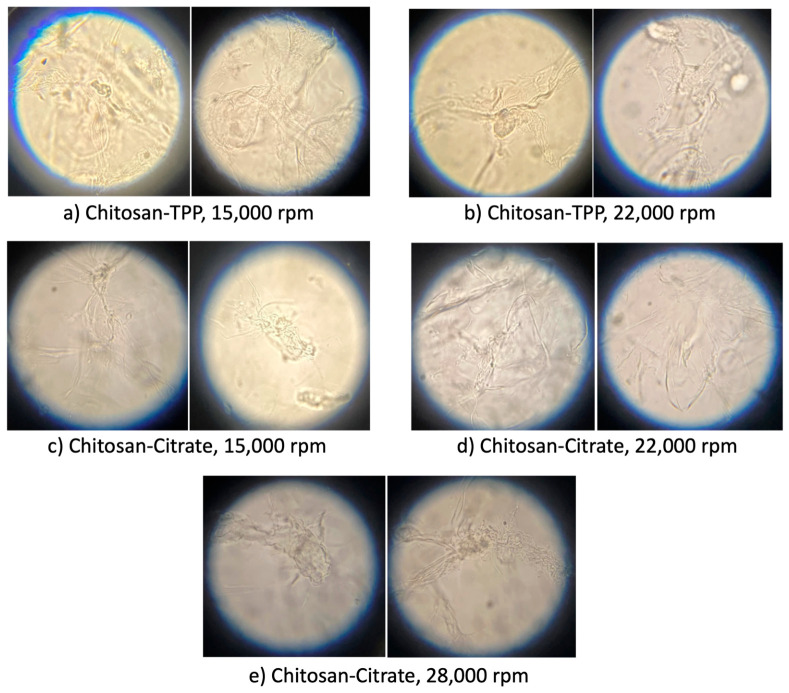
Optical micrographs of freshly precipitated chitosan particles prepared using TPP and citrate precipitation systems at different homogenization speeds: (**a**) chitosan–TPP, 15,000 rpm; (**b**) chitosan–TPP, 22,000 rpm; (**c**) chitosan–citrate, 15,000 rpm; (**d**) chitosan–citrate, 22,000 rpm; and (**e**) chitosan–citrate, 28,000 rpm. Two independent fields of view are shown for each condition to illustrate intra-sample heterogeneity and aggregate variability. Images were acquired immediately after particle preparation at 40× magnification.

Freshly precipitated chitosan particles exhibited irregular dendritic, fibrillar and flocculated aggregate morphologies under all investigated homogenization conditions (Figure 4). Chitosan–TPP particles prepared at 15,000 and 22,000 rpm retained visible branching and aggregated microgel-like domains, whereas chitosan–citrate systems prepared at 15,000–28,000 rpm showed irregular fibrillar and dendrite-like structures. Two independent optical fields of view are shown for each preparation condition in Figure 4 to illustrate intra-sample heterogeneity and aggregate variability without quantitative particle-size distributions. The observed morphologies are consistent with ionic-gelation systems in which local polyelectrolyte complexation, shear-induced fragmentation and secondary aggregation occur simultaneously [26,27,28].

**Figure 5:**
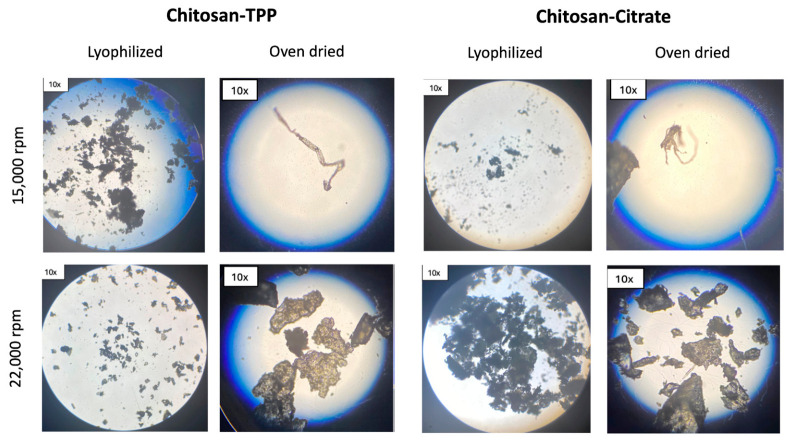
Optical micrographs of lyophilized and oven-dried chitosan particles prepared using TPP and citrate precipitation systems at homogenization speeds of 15,000 and 22,000 rpm. Images were acquired at 10× magnification. Lyophilized citrate- and TPP-precipitated particles retained more porous and flocculated aggregate structures, whereas oven-dried particles appeared denser and more compact.

The drying route exerted a stronger influence on the final particle architecture than the initial visual morphology. Lyophilized chitosan–citrate and chitosan–TPP particles formed porous flocculated aggregates with low apparent density, whereas convectively dried particles appeared denser, more compact and partially collapsed (Figure 5).

For citrate-precipitated particles, increasing the homogenization speed from 15,000 to 22,000 rpm resulted in larger and more compact agglomerates, suggesting enhanced collision-driven aggregation under high shear conditions. In contrast, TPP-precipitated particles processed at 22,000 rpm exhibited finer and more dispersed aggregate structures, consistent with stronger fragmentation of ionically crosslinked TPP-rich flocs. These opposite trends indicate that the effect of homogenization cannot be interpreted independently from counterion chemistry.

### 3.2. Mechanical Stability of the Composite Membranes Depends on Polymer Composition

The investigated polymer systems exhibited markedly different film-forming behavior depending on the polymer composition and plasticizer content. Agarose/glycerol and cellulose acetate/PEG-400 systems formed continuous and mechanically stable films, whereas several shellac-rich and alginate-containing formulations exhibited excessive brittleness, cracking or strong adhesion to the casting molds. The formulation map and representative membrane photographs indicate that film integrity was achieved through plasticizer addition and composite blending rather than through a single universal matrix.

Chitosan-containing composite systems generally showed improved film integrity and flexibility compared to the corresponding non-chitosan formulations. The addition of glycerol and PEG-400 reduced the dry-state rigidity of neat chitosan films and improved processability, although excessive plasticization also resulted in a pronounced decrease in puncture resistance. The combined glycerol/PEG-400 chitosan formulation showed a slight increase in puncture resistance compared to glycerol-only chitosan films, consistent with partial network reorganization induced by PEG-400.

**Figure 6:**
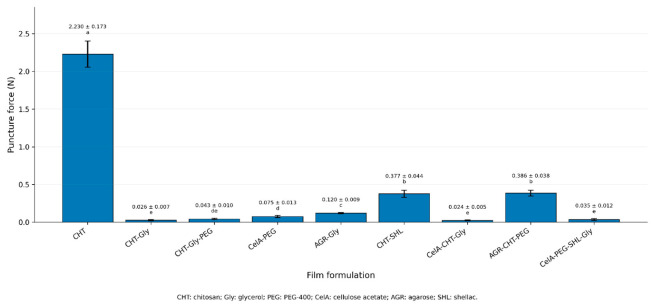
Dry-state penetrometric puncture force of the prepared biopolymer films. Error bars represent standard deviation. Different letter groups indicate statistically significant differences between formulations at *p* < 0.05. These measurements were used as an initial dry-state stress screen of film integrity and brittleness after drying and processing.

The comparative dry-state puncture-force measurements summarized in Figure 6 confirmed the strong influence of polymer composition on the mechanical behavior of the prepared membranes after drying. Neat chitosan exhibited the highest dry-state puncture resistance among all investigated systems, indicating a rigid and cohesive hydrogen-bonded polysaccharide network under dry conditions. In contrast, highly plasticized formulations displayed substantially lower puncture-force values and weaker resistance to localized mechanical loading.

Among the investigated composite systems, chitosan/shellac and agarose/chitosan/PEG-400 formulations exhibited the highest dry-state mechanical resistance, suggesting improved structural compatibility between the blended polymer phases. In contrast, cellulose acetate/chitosan/glycerol and highly plasticized systems remained mechanically weak despite successful film formation. Cellulose-acetate-containing systems may therefore be more suitable as supporting or backing layers within future multilayer membrane architectures rather than as standalone candidate immobilization interfaces under the present formulation conditions.

### 3.3. EDC/NHS Reactivity Is Dominated by Chitosan and Shellac Functionality

The dry-state mechanical screening alone was insufficient to identify the most suitable candidate immobilization support for enzymatic DAC applications, because EDC/NHS coupling efficiency depends primarily on the availability of reactive amine- and carboxyl-containing functional groups. The investigated membrane systems were therefore comparatively evaluated with respect to their expected chemical reactivity, hydrated accessibility and mechanical integrity. The resulting functional assessment is summarized in Table 2.

The evaluation indicated that chitosan-containing systems exhibited the highest expected EDC/NHS reactivity due to the presence of primary amine groups capable of participating in covalent coupling reactions. Shellac-containing systems were also considered chemically favorable because shellac contributes carboxyl functionalities that can be activated through EDC/NHS chemistry. In contrast, cellulose-acetate-rich systems primarily contributed film-forming and structural characteristics, while their direct participation in EDC/NHS-mediated immobilization was expected to be limited. Agarose-based systems were considered particularly relevant for maintaining hydrated polysaccharide environments potentially favorable for enzyme stabilization, although agarose itself was not expected to contribute strongly to covalent coupling chemistry.

**Figure 7:**
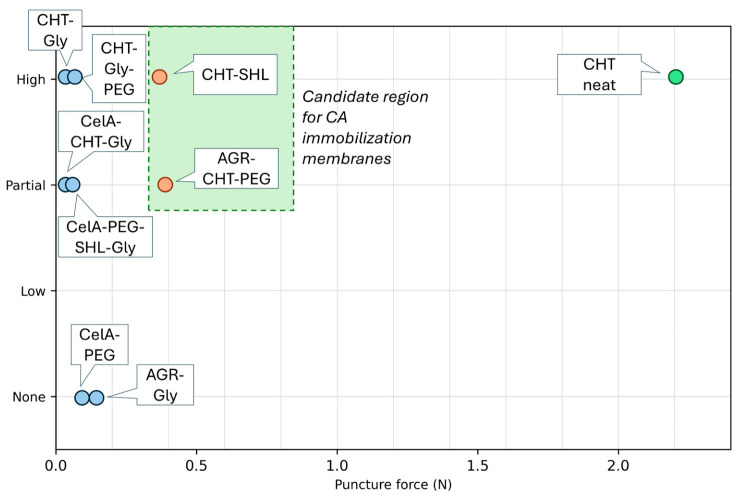
Selection matrix correlating dry-state membrane puncture resistance with the expected EDC/NHS reactivity of the investigated polymer systems. Abbreviations: CHT—chitosan; Gly—glycerol; PEG—PEG-400; CelA—cellulose acetate; AGR—agarose; SHL—shellac.

The multi-criteria selection matrix presented in Figure 7 integrates the mechanical and chemical screening results in order to identify the most promising membrane candidates for carbonic anhydrase immobilization. The shaded region highlights the preferred balance between dry-state puncture resistance and expected EDC/NHS reactivity for future immobilization-support validation. Chitosan/shellac (CHT-SHL) emerged as the most balanced composite formulation because it combines relatively high dry-state puncture resistance with the simultaneous presence of amine-rich chitosan domains and shellac-derived carboxyl functionalities. Agarose/chitosan/PEG-400 (AGR-CHT-PEG) also exhibited favorable positioning within the target region due to its intermediate mechanical stability and hydrated polysaccharide character.

Neat chitosan (CHT) exhibited the highest dry-state puncture resistance among all investigated systems and therefore remains an important reactive mechanical reference in the present screen. Dry puncture resistance alone is not sufficient to determine whether neat chitosan is appropriate as a standalone hydrated immobilization support. Therefore, neat chitosan is retained here as a reference material, while chitosan/shellac and agarose/chitosan/PEG-400 are identified as more balanced candidate composites on the basis of the current dry-state puncture, formulation and expected-reactivity screen.

### 3.4. FTIR Spectroscopy Reveals Functional-Group Changes After EDC/NHS Treatment

**Figure 8:**
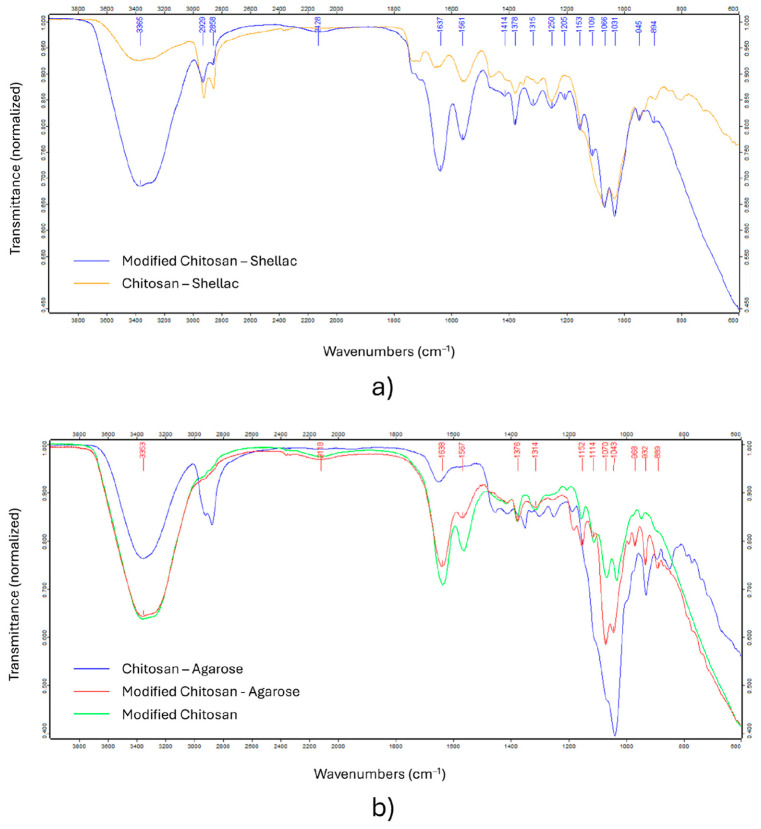
Representative FTIR spectra of selected polymer membranes before and after EDC/NHS treatment: (**a**) chitosan/shellac and (**b**) chitosan/agarose systems.

FTIR spectroscopy was performed on selected polymer membranes before and after EDC/NHS treatment in order to evaluate changes in functional groups relevant to immobilization chemistry. The chitosan/shellac and chitosan/agarose systems were selected for detailed presentation because they demonstrated the most favorable balance between mechanical stability, hydrated compatibility and expected EDC/NHS reactivity during the comparative screening described in Section 3.2 and Section 3.3. Representative spectra of these systems are shown in Figure 8, while additional spectra of cellulose-acetate-containing formulations are provided in Supplementary Figure S1. These spectra were used only to assess EDC/NHS-associated polymer-support modification, not as evidence of carbonic anhydrase immobilization.

The chitosan/shellac system exhibited the most pronounced spectral modifications after EDC/NHS treatment, thus providing the strongest polymer-support evidence for the intended candidate design (Figure 8a). Blending broadened the O–H/N–H region, introduced shellac-associated carboxyl/ester carbonyl features and modified the 1300–1000 cm^−1^ fingerprint region. After EDC/NHS treatment, free-carboxyl intensity decreased, amide-associated intensity in the 1650–1600 cm^−1^ range increased, and C–N/amide-III-related contributions in the 1300–1200 cm^−1^ region changed. These shifts are consistent with EDC/NHS-mediated activation of shellac carboxyl groups and formation of new amide-like or activated-ester polymer environments at chitosan/shellac interfaces [21,22,23,24,41,42,43].

The chitosan/agarose system also demonstrated detectable spectral redistribution after modification (Figure 8b), particularly within the hydroxyl-rich stretching region and the polysaccharide fingerprint domain. Agarose/glycerol and chitosan/agarose spectra were dominated by broad O–H/N–H stretching between 3600 and 3200 cm^−1^, water-associated bending near 1640 cm^−1^ and strong polysaccharide C–O/C–O–C vibrations between 1150 and 1030 cm^−1^. The chitosan-containing agarose composite showed additional amide/amine contributions, and EDC/NHS modification caused narrowing or redistribution in the O–H/N–H envelope and changes in amide-associated regions. This supports the assignment of chitosan as the reactive component and agarose as a hydrated structural partner.

Supplementary Figure S1 presents the FTIR spectra of cellulose acetate/shellac (Figure S1a) and cellulose acetate/chitosan (Figure S1b) systems before and after EDC/NHS treatment. Representative FTIR band assignments and spectral interpretations for the cellulose-acetate-containing systems are summarized in Supplementary Table S2. Both cellulose-acetate-containing formulations exhibited characteristic ester-associated carbonyl and C–O/C–O–C vibrations typical for cellulose acetate matrices. The shellac-containing system showed spectral redistribution consistent with the selective modification of shellac-rich domains, whereas the chitosan/cellulose acetate system exhibited changes associated primarily with chitosan-rich or interfacial regions after treatment. In both cases, the cellulose-acetate ester backbone remained comparatively stable, supporting the interpretation that cellulose acetate contributes primarily to structural and film-forming functionality within the investigated membrane systems.

## 4. Discussion

The results support a specific design principle for candidate enzymatic DAC membranes: the film should balance reactive functionality, hydrated accessibility and sufficient mechanical integrity. Neat chitosan established the upper dry-state mechanical bound of the screen and is retained as a reactive reference material, while plasticizer addition and polymer blending improved processability. This explains why chitosan/shellac and agarose/chitosan/PEG-400 are more balanced candidate composites than neat chitosan within the present support-selection stage.

The membranes should be treated as microphase-heterogeneous biopolymer composite films in which local polymer-rich domains, particle aggregates and drying-induced concentration gradients may coexist. Such heterogeneity is not necessarily incompatible with enzyme-support development, because hydrated domains and local roughness may increase accessible surface area; however, uncontrolled heterogeneity may reduce reproducibility, generate weak mechanical points and lead to nonuniform enzyme loading. Future optimization should therefore focus on solvent compatibility, controlled mixing order, reduced shellac loading, longer high-shear or ultrasonic dispersion, degassing before casting, thinner film casting and bilayer or multilayer architectures in which a reactive chitosan/shellac-rich layer is supported by a mechanically continuous backing layer.

Chitosan/shellac is the lead candidate formulation because its chemistry directly matches the EDC/NHS coupling mechanism. Shellac contributes carboxyl groups that can be activated by EDC/NHS, while chitosan provides amine-rich domains and a hydrophilic polysaccharide environment. The FTIR shifts observed after modification occur in the expected carbonyl, amide and C-O/C-N regions, supporting a chemically meaningful support-activation process. Mechanically, chitosan/shellac retained substantially higher puncture resistance than the weak cellulose-acetate blends, making it the strongest chemical-mechanical compromise in the dataset.

Agarose/chitosan/PEG-400 is a second rational candidate, but for a different reason. Its puncture force was slightly higher than that of chitosan/shellac, and its hydrated polysaccharide matrix may help maintain the aqueous microenvironment required for CA catalysis. However, agarose itself is not the main EDC/NHS-reactive component; reactivity is supplied by chitosan. This formulation is therefore best interpreted as a hydration-rich comparator rather than the most direct chemical coupling platform. The comparative strengths, limitations and proposed functional roles of the leading membrane systems within the enzymatic DAC concept are summarized in Table 3.

Cellulose-acetate-containing systems should be assigned a limited but still useful role in the present design space. Cellulose acetate provides bio-derived film-forming capacity and compatibility with membrane fabrication, but its ester-rich backbone is not the main source of EDC/NHS-reactive functionality. In the present formulations, the reactive contribution comes primarily from chitosan or shellac fractions. Therefore, cellulose acetate is more appropriately considered as a potential structural or backing-layer component in future multilayer membrane architectures, while chitosan/shellac-rich domains provide the more direct enzyme-coupling chemistry. Polymers, such as PAA, PMAA and polyamide-based materials, remain important synthetic or semi-synthetic benchmarks for future comparisons when immobilization efficiency becomes the primary selection criterion.

The particle results show that upstream processing can tune the physical form of the chitosan phase before film formation. Lyophilized chitosan particles preserved porous, flocculated architectures that are likely to increase surface accessibility and swelling, whereas convective drying produced denser structures that may reduce accessible area but improve compactness. The divergent response of citrate and TPP systems to increasing homogenization speed suggests that precipitation chemistry must be fixed before any scale-up or comparative enzyme-loading study. The present optical microscopy should therefore be interpreted as qualitative morphology screening; DLS, SEM and cross-sectional imaging are necessary future experiments for resolving particle-size distribution, dispersion inside the matrix and through-thickness membrane architecture.

The FTIR data should be interpreted as evidence of EDC/NHS-associated chemical modification of the polymer supports rather than proof of carbonic anhydrase immobilization. Since carbonic anhydrase was not present during these experiments, no spectral band can be assigned to immobilized enzyme, retained protein structure or support-enzyme bond formation. The observed changes in the carbonyl, amide-associated and C-O/C-O-C regions instead support activation- or crosslinking-related changes within chitosan- and shellac-containing polymer domains.

For membrane DAC, these materials can be positioned as biological hydration-promoting membranes. Their role is to present immobilized CA in a hydrated and chemically stable microenvironment where CO_2_ entering from the gas phase or a gas-liquid contact interface can be rapidly converted to bicarbonate. Gas enrichment, product separation, sorbent regeneration and concentration of the final CO_2_ stream require additional process units or membrane layers beyond the present materials screen.

The present study defines the support-selection stage of polymer carriers rather than a completed enzymatic CO_2_-capture process. Direct CA immobilization, immobilized protein loading, retained activity, leaching resistance, wet-state puncture resistance, swelling ratio, contact angle, tensile or dynamic mechanical behavior, reusability and CO_2_ hydration or absorption kinetics remain necessary validation metrics. Bradford or BCA assays can quantify immobilized protein [44,45]. Wilbur–Anderson or colorimetric hydration assays can quantify CA activity [46,47].

## 5. Conclusions

This study establishes a first-stage screening framework for EDC/NHS-reactive biopolymer composite membranes intended as candidate carbonic anhydrase immobilization supports for enzymatic direct air capture. Among the tested formulations, chitosan/shellac provided the most favorable balance between dry-state puncture resistance and expected reactive functionality, while agarose/chitosan/PEG-400 served as a hydration-rich comparator with intermediate mechanical stability. Cellulose-acetate-containing systems were less suitable as standalone reactive supports under the present formulation conditions but remain relevant as possible structural or backing layers in multilayer membrane architectures.

Direct enzyme loading, retained catalytic activity, leaching resistance, wet-state mechanical stability, swelling behavior, contact angle, reusability and CO_2_ hydration or absorption kinetics are required before these membranes can be described as validated enzymatic DAC units.

## Figures and Tables

**Table 1:** Functional role of the membrane components used in the biopolymer composite screen.

Component	Primary Function in the Membrane Screen	Main Reactive/Structural Contribution
Chitosan	Reactive polysaccharide support and particle precursor	Primary amines, hydroxyls, hydrogen-bonding network, ionic gelation with TPP/citrate
Shellac	Carboxyl-bearing resinous co-component	Carboxylic acid groups for EDC/NHS activation; hydrophobic/hydrogen-bonding domains
Agarose	Hydrated polysaccharide network former	Hydroxyl-rich gel network; hydration and matrix continuity; low direct EDC/NHS reactivity
Cellulose acetate	Film-forming structural polymer	Ester-rich membrane-forming matrix; limited direct EDC/NHS reactivity
Glycerol	Small-molecule plasticizer	Increased flexibility and hydrophilicity; reduced cohesive strength at high plasticizer content
PEG-400	Polyether plasticizer/ co-plasticizer	Chain mobility control, spacing, hydrogen-bond modulation and porosity effects

**Table 2:** Qualitative EDC/NHS reactivity map of the film classes.

Film	EDC/NHS Reactivity	Chemical Basis and Interpretation
Chitosan	Reactive	Primary amines provide coupling-relevant functionality.
Cellulose acetate	Non-reactive	No directly available amine/carboxyl pair for zero-length amidation.
Shellac	Reactive	Carboxyl groups provide EDC/NHS-activatable sites.
Agarose	Non-reactive	Hydroxyl-rich matrix without direct amine/carboxyl coupling sites.
Chitosan + cellulose acetate	Partially reactive	Reactivity derives from chitosan-rich domains.
Shellac + cellulose acetate	Partially reactive	Reactivity derives from shellac-derived carboxyl groups.
Chitosan + shellac	Highly reactive	Both amine and carboxyl functionalities are present in the same composite.
Chitosan + agarose	Partially reactive	Chitosan provides amine functionality; agarose contributes hydration and structure.

**Table 3:** Interpretation of the leading support candidates for enzymatic DAC membrane development.

Candidate	Strengths	Weaknesses	Proposed Role in Enzymatic DAC
Chitosan/shellac	High EDC/NHS relevance; balanced puncture resistance; FTIR evidence of shellac/chitosan modification	Possible microphase heterogeneity and handling sensitivity	Lead candidate support for CA immobilization
Agarose/chitosan/PEG-400	Highest composite puncture force; hydrated polysaccharide environment	EDC/NHS chemistry mostly derives from chitosan, not agarose	Hydration-rich comparator and alternative biocatalytic support
Neat chitosan	Highest puncture force; abundant amines	Dry-state rigidity, potential stiffness, brittleness and swelling dependence	Reactive dry-state mechanical reference
Cellulose acetate composites	Useful processability and membrane-forming background	Low puncture force and limited intrinsic EDC/NHS reactivity	Potential structural or backing layer in multilayer membranes

## Data Availability

The original contributions presented in this study are included in the article/Supplementary Materials. Further inquiries can be directed to the corresponding authors.

## Supplementary Materials

The following supporting information can be downloaded at: https://www.mdpi.com/article/10.3390/ma19132869/s1, Table S1: Detailed compositions of the investigated polymer membrane formulations; Figure S1: Representative FTIR spectra of selected polymer membranes before and after EDC/NHS treatment: (a) chitosan/cellulose acetate and (b) shellac/cellulose acetate. Table S2. Representative FTIR band assignments and interpretation of cellulose-acetate-containing membrane systems before and after EDC/NHS treatment.

## Abbreviations

The following abbreviations are used in this manuscript:

EDC/NHS	N-(3-dimethylaminopropyl)-N′-ethylcarbodiimide hydrochloride/N-hydroxysuccinimide
DAC	Direct air capture
CA	Carbonic anhydrase
PEG-400	Polyethylene glycol 400
FTIR	Fourier-transform infrared spectroscopy
TPP	Sodium tripolyphosphate
ATR	Attenuated total reflection
CHT	Chitosan
Gly	Glycerol
CelA	Cellulose acetate
AGR	Agarose
SHL	Shellac
