# Supplementary material for: Candidate Biopolymer Composite Membranes for Carbonic Anhydrase Immobilization in Enzymatic Direct Air Capture
Source: Materials (Basel). 2026 Jul 5;19(13):2869. doi: 10.3390/ma19132869 (PMC13363688; doi:10.3390/ma19132869)
Supplement: Supplementary file 1 [file materials-19-02869-s001.zip › materials-4365792-supplementary.pdf]

Candidate Biopolymer Composite Membranes for Carbonic Anhydrase Immobilization in Enzymatic Direct Air Capture

Supplementary Materials

Spas Kerimov [1,*], Victoria Atanassova [1], Georgi Yankov [1,*], Radostin Stefanov [1], Ekaterina Iordanova [1], Georgi Marinov [2], Hristo Kalaydzhiev [3] and Albert Krastanov [4]

Contents

- **Table S1.** Detailed compositions of the investigated polymer membrane formulations.
- **Figure S1.** Representative FTIR spectra of selected polymer membranes before and after DC/NHS treatment: (a) chitosan/cellulose acetate and (b) shellac/cellulose acetate.
- **Table S2.** Representative FTIR band assignments and interpretation of cellulose-acetate-containing membrane systems before and after EDC/NHS treatment.

Table S1. Detailed compositions of the investigated polymer membrane formulations.

No.	Film formulation
CHT	Neat chitosan in acetic acid
CHT-Gly	Chitosan + glycerol
CHT-Gly-PEG	Chitosan + 10% glycerol + 10% PEG-400
CelA-PEG	Cellulose acetate + PEG-400
AGR-Gly	Agarose:glycerol (1:5), diluted to 20 mL with water
CHT-SHL	Chitosan (1.5%) + shellac (5% in 95% ethanol), ethanol:chitosan 1:2
CelA-CHT-Gly	Cellulose acetate + chitosan + glycerol
AGR-CHT-PEG	Agarose in glycerol + chitosan in acetic acid + PEG-400
CelA-PEG-SHL-Gly	Cellulose acetate + PEG-400 + shellac + glycerol

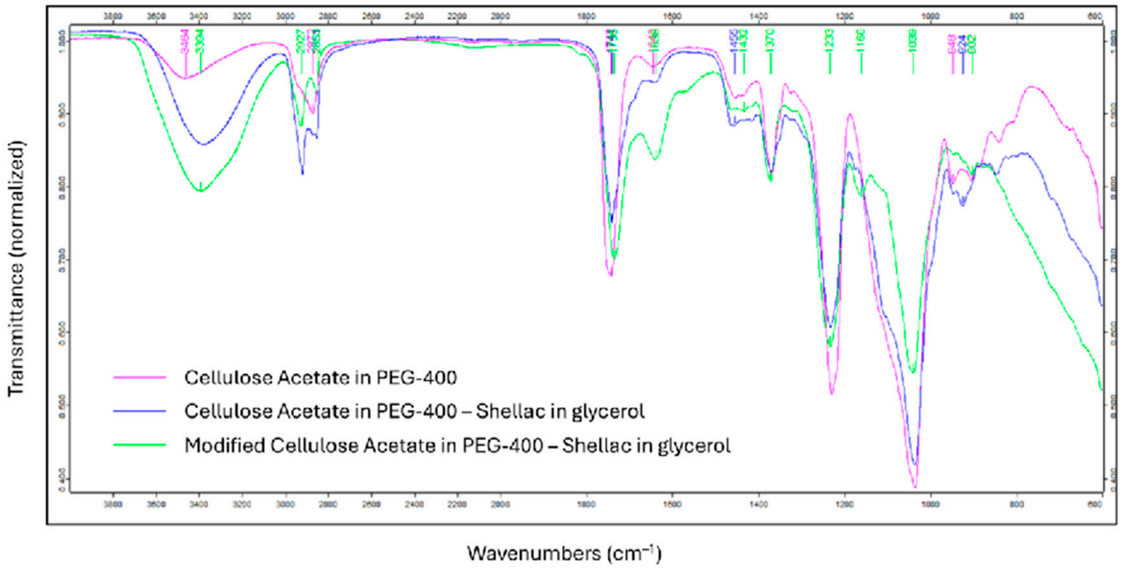

b)

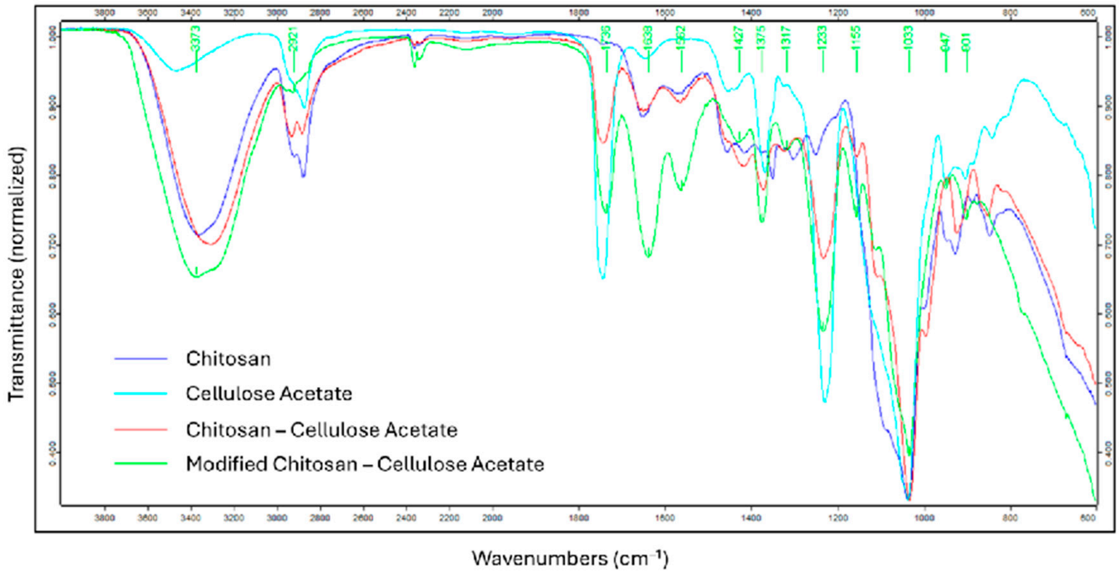

a)

Figure S1. Representative FTIR spectra of selected polymer membranes before and after EDC/NHS treatment: (a) chitosan/cellulose acetate and (b) shellac/cellulose acetate.

Table S2. Representative FTIR band assignments and interpretation of cellulose-acetate-containing membrane systems before and after EDC/NHS treatment.

Polymeric System	Spectral feature (cm^{-1})	Assignment	Interpretation after EDC/NHS treatment
Cellulose acetate/PEG-400	3400	O–H stretching	PEG-associated hydroxyl contribution
	2940–2880	Aliphatic C–H stretching	Polymer backbone vibrations
	1735–1745	Ester carbonyl (C=O)	Characteristic cellulose acetate ester band
	1230–1245	Ester C–O–C stretching	Acetylated cellulose structure
Cellulose acetate/shellac	1710–1720	Carboxylic-acid carbonyl	Shellac-derived carboxyl groups
	1550–1600	Amide/activated-group region	Redistribution after EDC/NHS treatment
	1200–1300	C–O/C–N region	Consistent with selective modification of shellac-rich domains
Cellulose acetate/chitosan	3400	O–H/N–H stretching	Chitosan hydroxyl and amine contributions
	1650	Amide I	Chitosan-associated band
	1580	Amide II/free amine	Chitosan-rich/interfacial-domain contribution
	1240–1030	C–O/C–O–C stretching	Mixed polysaccharide and ester vibrations
